# Differences in the evolutionary history of disease genes affected by dominant or recessive mutations

**DOI:** 10.1186/1471-2164-7-165

**Published:** 2006-07-03

**Authors:** Simon J Furney, M Mar Albà, Núria López-Bigas

**Affiliations:** 1 Genome Bioinformatics Laboratory. Centre for Genomic Regulation, Universitat Pompeu Fabra, Pg. Maritim de la Barceloneta 37-49, E-08003, Barcelona, Spain; 2 ICREA – Institut Municipal d'Investigació Mèdica. Universitat Pompeu Fabra, Dr. Aiguader 80, 08003, Barcelona, Spain; 3 Conway Institute of Biomolecular and Biomedical Research, University College Dublin, Belfield, Dublin 4, Ireland

## Abstract

**Background:**

Global analyses of human disease genes by computational methods have yielded important advances in the understanding of human diseases. Generally these studies have treated the group of disease genes uniformly, thus ignoring the type of disease-causing mutations (dominant or recessive). In this report we present a comprehensive study of the evolutionary history of autosomal disease genes separated by mode of inheritance.

**Results:**

We examine differences in protein and coding sequence conservation between dominant and recessive human disease genes. Our analysis shows that disease genes affected by dominant mutations are more conserved than those affected by recessive mutations. This could be a consequence of the fact that recessive mutations remain hidden from selection while heterozygous. Furthermore, we employ functional annotation analysis and investigations into disease severity to support this hypothesis.

**Conclusion:**

This study elucidates important differences between dominantly- and recessively-acting disease genes in terms of protein and DNA sequence conservation, paralogy and essentiality. We propose that the division of disease genes by mode of inheritance will enhance both understanding of the disease process and prediction of candidate disease genes in the future.

## Background

Understanding the genetic basis of human inherited disorders is one of the primary goals of medical genetics. By applying the knowledge gleaned from this research, disease prevention and treatment can be improved. Currently, there are more than 1600 human genes known to be associated with particular Mendelian disease phenotypes. The analysis of this group of genes from a global perspective has already revealed interesting insights about the nature of human disease [1-5].

Several reports have analysed the conservation pattern of disease genes compared to the rest of genes [2-7]. Human disease genes have been found to be more conserved at the protein level, in general, than the remainder of the human proteome [2], and most have been found to possess homologues in the mouse and rat genomes [3]. Furthermore, a study of human genes involved in disease found lower non-synonymous substitution rate (K_A_) in the disease genes compared to generic genes [5]. Another study of sequence conservation at the nucleotide level between human and rat found only a small difference between the K_A_/K_S _ratio of human disease genes and non-disease genes, although a significantly more elevated synonymous substitution rate (K_S_) in the disease genes was discovered [3]. Analysis of the chimpanzee genome also found a higher synonymous substitution rate in disease genes that was attributed to a higher relative frequency of CpG dinucleotides [6]. Recently another work reported the analysis of human-rodent K_A_/K_S _ratios of disease genes compared to housekeeping and the rest of genes, finding that housekeeping genes are the most conserved group with disease genes showing intermediate values between the housekeeping and other genes [7].

These studies have treated the disease genes as homogeneous datasets and in doing so have neglected an inherent difference within the sets of genes, namely the molecular genotype underlying the disease state. Many autosomal Mendelian disease phenotypes can be understood in terms of dominant and recessive mutations affecting a particular gene. In cases in which the mutation is said to be dominantly-acting, a mutation to a single allele of the gene is sufficient to cause the disease state. Genes susceptible to disease-causing mutations in this way are often haploinsufficient. The mutation can cause loss of function of the protein by dominant negative effects, or reduced or compromised functioning. Diseases caused by recessive mutations require both alleles to be affected, as the production of a functional protein from one allele is often enough to satisfy physiological requirements (haplosufficiency).

The phenomenon of dominance and recessitivity in diploid organisms was first recorded quantitatively by Gregor Mendel in 1866 [8], and the evolution of dominance has been a topic of debate between geneticists for the last century [9]. Fisher, in 1928, reported that most mutations observed in Drosophila were recessive to the wild-type alleles [10]. He attributed this to a balance between recurrent mutations and their elimination by natural selection. However, Wright (1929) challenged this hypothesis stating that unrealistically high levels of selective pressure were necessary for the evolution of dominance in this manner [11]. He proposed that dominance was an inherent consequence of physiology. Kascer and Burns (1981) developed a metabolic model for dominance congruent with Wright's physiological model [12]. However, the metabolic model has been criticised because it is solely based on the kinetic properties of metabolic enzymes [13].

Previous studies have analysed human disease genes in terms of the nature of the mutation underlying the disease phenotype [1,2,5]. However the focus of these studies has been mainly on differences in the functional classification of dominant and recessive disease genes. Jimenez-Sanchez *et al*. showed that diseases caused by genes coding for enzymes were predominantly recessive, whereas disorders in which the causative gene coded for a transcription factor were, in the main, dominantly-acting [1]. Lopez-Bigas *et al*. have reported similar results and further demonstrate that dominant human disease genes have closer paralogues than recessive disease genes [14]. Kondrashov and Koonin reported that haploinsufficient (dominant) disease genes have more paralogues in the human genome than haplosufficient (recessive) genes [15].

However, none of these studies has investigated differences in sequence conservation between disease genes depending on their mode of inheritance. Smith and Eyre-Walker [4] analyse gene evolution in the Jimenez-Sanchez *et al*. dataset and report higher conservation in dominant disease genes compared to recessive disease genes, but this study also observes less selective constraints on disease genes compared to non-disease genes, a result that is contrary to evidence from other reports, and could be due to the small number of genes analysed [5].

We have undertaken a comprehensive study of the molecular evolution of the autosomal human disease genes depending on their mode of inheritance; namely genes affected by dominant mutations and by recessive mutations. We have investigated differences in coding sequence divergence, protein conservation, human paralogy, *C. elegans *lethality, protein function, gene structure and severity of disease. The comparison of the evolutionary patterns of dominant and recessive disease genes reveals important differences between these two sets of genes that can be understood in terms of their different hereditary nature, giving further insights into the understanding of hereditary human diseases.

## Results

### Differences in level of protein conservation between disease proteins affected by dominant or recessive mutations

**Figure 1:**
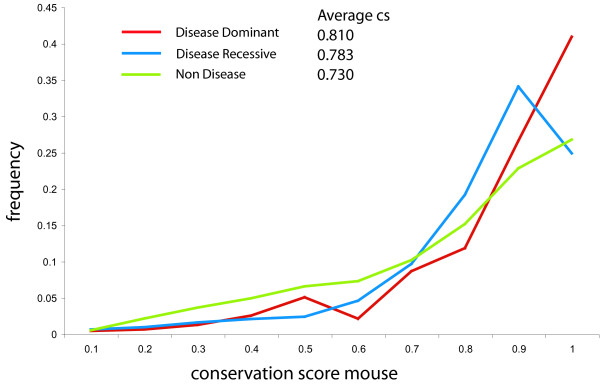
**Conservation of dominant and recessive disease genes at protein level. **Distribution of conservation score in mouse of dominant and recessive disease genes versus the rest of genes.

The division of disease genes reveals that depending on the mode of inheritance the level of conservation of the protein is different. Human proteins encoded by genes affected by dominant mutations are more conserved in mouse than those encoded by genes affected by recessive mutations (p-value for Mann-Whitney (M-W) test = 7.59 × 10^-6^; Tables 1 &2). When we plot the frequency distributions of the protein conservation scores (cs, see Methods) of the different sets of genes, we observe that the set of dominant disease genes (DD) has a significantly different distribution to recessive disease genes (DR) (p-value for Kolmogorov-Smirnov (K-S) test = 1.95 × 10^-6^, Table 2 and Figure 1) and both sets of disease genes are significantly different to the non-disease genes (p-value for both K-S tests < 2.2 × 10^-16^). Recessive disease genes display a sharp decrease in frequency at high levels of protein conservation scores (cs > 0.9), a trend not apparent in dominant disease or non-disease genes. Consistent results are observed with the analysis of cs in other vertebrates, namely *Rattus norvegicus*, *Gallus gallus*, *Takifugu rubripes*, and *Danio rerio *[see Additional file 1].

### Level of protein conservation of paralogues of dominant and recessive disease genes

**Figure 2:**
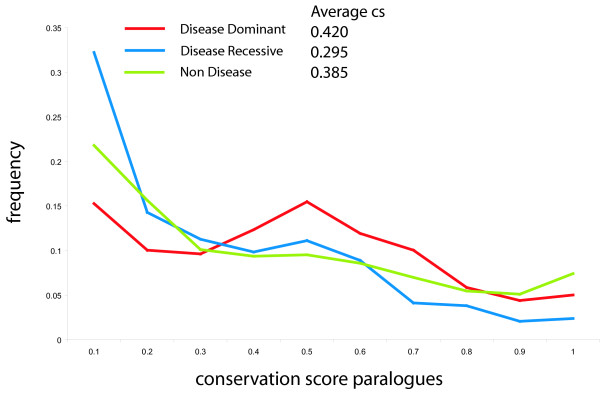
**Conservation of paralogues of dominant and recessive disease genes at protein level. **Distribution of conservation score in paralogues of dominant and recessive disease genes versus the rest of genes.

Previously, it has been reported that genes involved in disease have less conserved paralogues than human genes in general [2], presumably because highly similar paralogues can potentially compensate for a mutated protein [16], in which case a disease might not be observed. This is confirmed by our analysis, in which the average conservation score (cs) of paralogues is 0.349 for human disease genes and 0.385 for non-disease genes (p-value for M-W test = 5.24 × 10^-4^, Tables 1 &2). However, by classifying the disease genes by the mode of inheritance of the phenotype, we can see that significant differences are observed between genes involved in dominant or recessive diseases (p-value for M-W test = 4.44 × 10^-16^; Tables 1 and 2), in congruence with previous results [14]. The average cs value of paralogues of recessive disease genes is 0.295, while that of dominant disease genes is 0.420, even higher than for the non-disease genes. However, when we examine the sequence conservation pattern of paralogues more closely, we observe that non-disease genes have a higher proportion of highly similar paralogues (cs > 0.8) than either of the disease gene groups (Figure 2). This difference is more pronounced in recessive than in dominant disease genes. To assess these differences statistically we calculated Z-scores and p-values for dominant and recessive disease genes against 10,000 randomly generated sets of proteins. Recessive disease genes have a significantly lower than expected number of highly conserved paralogues (Z-score = -2.76, p-value = 2 × 10^-3 ^for cs > 0.8). The number of dominant disease gene paralogues with cs > 0.8 is, however, not significantly different to that expected by random sampling of the proteins. In conclusion, the lower number of less conserved paralogues in disease versus non-disease genes can be entirely attributed to the recessive disease genes.

### Selective pressures acting on dominant and recessive disease genes at the DNA level

**Figure 3:**
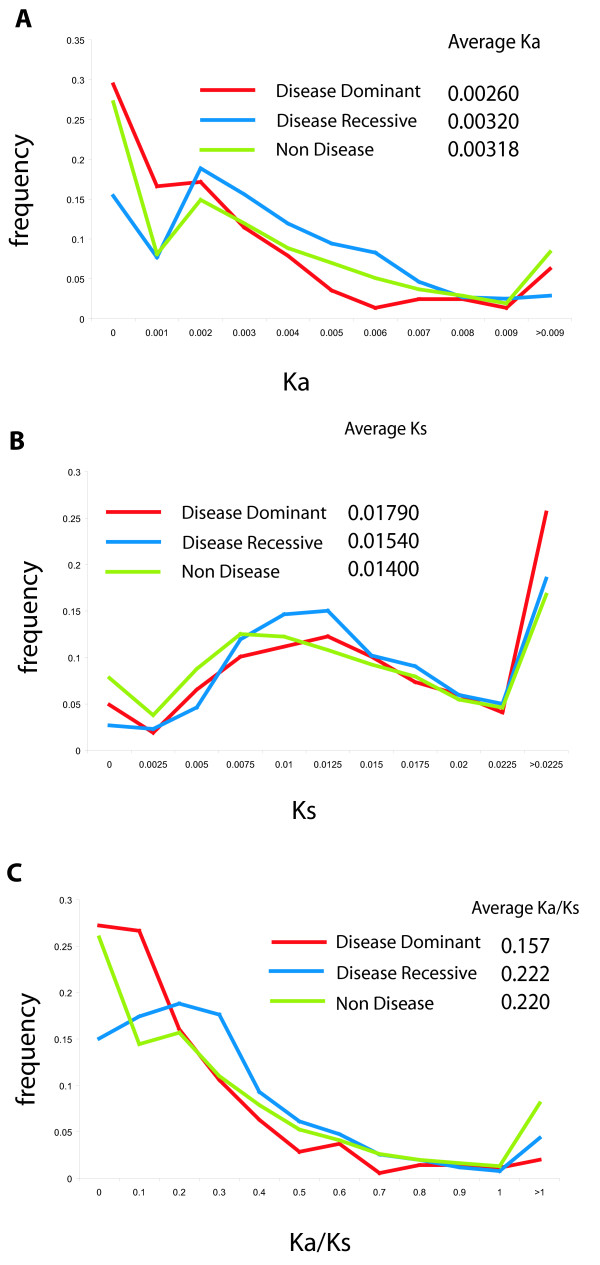
**Evolutionary rates between human and chimpanzee genes of dominant and recessive disease genes at DNA level. **(A) Distribution of K_A _for disease genes versus the rest of genes. (B) Distribution of K_S _for disease genes versus the rest of genes. (C) Distribution of K_A_/K_S _ratio of disease genes versus the rest of genes.

We analysed the level of conservation at the DNA level of these sets of genes in order to obtain a better understanding of the selective pressures acting on them. We examined non-synonymous (K_A_) and synonymous (K_S_) coding sequence substitution rates, as well as intron sequence substitution rates (K_I_), from human-chimpanzee orthologues [6]. K_A _is indicative of the selective pressure acting on sites that involve a change of amino acid, while K_S _is more reflective of the background mutation level. Disease genes taken together show lower K_A _values than non-disease genes (average K_A _0.00295 vs. 0.00318, Table 3), in agreement with previous results [5]. However, the strongest differences are found between the two types of disease genes (M-W test p-value = 7.17 × 10^-11^), with an average K_A _of 0.0026 for dominant disease genes and of 0.0032 for recessive disease genes (Table 3, Figure 3A). Dominant disease genes are thus evolving more slowly, in general, than recessive disease genes or non-disease genes, which indicates that they are subjected to stronger selective constraints. Interestingly, although the average value of K_A _between disease recessive and non-disease genes is very similar (0.00320 and 0.00318), the distribution of K_A _values for these two sets is clearly different (p-value for K-S test = 2.66 × 10^-10 ^and Figure 3A). In fact, we observe a much lower proportion of disease recessive genes with very low K_A _value (<0.001) while intermediate K_A _values are over-represented in this group of genes (Figure 3). This is in agreement with the observed distribution of conservation scores (Figure 1) in which an under-representation of recessive disease genes is observed for very high conservation scores.

Disease genes show higher K_S _values, for both dominant and recessive genes, than the rest of human genes (p-value for M-W test disease versus non-disease = 8.34 × 10^-7 ^Figure 3B, Table 3). These differences could be ascribed to varying mutation rates in the genome, however the similarity in mean K_I _values (Table 3) and non-significant differences for the Mann-Whitney test (Table 2) for all groups would seem to negate this. Huang *et al*. reported a significantly elevated K_S _level in human disease genes compared to non-disease genes in an analysis between human and rat genes [3]. This phenomenon was also discovered in the analysis of the chimpanzee genome [6].

Due to the differences observed in K_S _between disease and non-disease genes it is necessary to analyse any differences in the ratio K_A_/K_S _between these groups of genes. This analysis confirms that, as observed in the conservation score and K_A _analyses, dominant disease genes exhibit the lowest evolutionary rates (Figure 3C, Table 3), while disease recessive genes have a similar average K_A_/K_S _value to the rest of genes (nD), although the distribution of K_A_/K_S _values between these two groups of genes is again very different (p-value for K-S test < 2.2 × 10^-16^).

Consistent results are found in the analysis of K_A_, K_S_, and K_A_/K_S _in human-mouse-dog orthologues [see Additional file 2].

### Essentiality in dominant and recessive disease genes

Our analysis shows that recessive disease genes are under-represented among highly conserved genes. One possibility is that in highly conserved proteins the occurrence of double mutations, as observed in recessive disease genes, is often associated with lethality, in which case a disease condition will not be observed. To assess this we mapped the genes from our study to *C. elegans *gene orthologues that have been previously classified as Wild Type (WT), disease (D) or Lethal (L) according to RNAi data [17]. Genes that are lethal when disrupted are considered "essential genes".

Firstly we determined that essential genes are significantly more conserved than WT genes (cs human-mouse: WT = 0.80, L = 0.84, p-value M-W test = 6.79 × 10^-6^; K_A _human-chimpanzee orthologues: WT = 0.0019, L = 0.0013, p-value M-W test = 2.19 × 10^-8^). The next question was whether recessive disease genes were under-represented among essential genes. The data show that the set of recessive disease genes has a lower proportion of *C. elegans *essential genes (31%) than the set of dominant disease genes (36%). In addition, only 47% of dominant disease orthologues have no phenotypic effect (WT) when mutated, in comparison to 61% of recessive disease orthologues and 58% of non-disease gene orthologues.

### Evolutionary conservation rates by functional annotations

Previously it has been found that the proportions of different functional annotations in dominant and recessive disease genes are not the same [1,14,15]. Diseases caused by mutations in genes coding for enzymes and transporters are mostly recessive, while mutations in transcription regulators, structural molecules, nucleic acid binding genes and signal transducers are primarily dominant. Therefore, it could be that the differences in evolutionary rates between dominant and recessive disease genes are due to the dissimilarities between the different types of functional genes and not due to distinct evolutionary histories of these two sets of genes. In order to rule out this possibility each human disease gene was classified according to the molecular function of its protein product as determined by Gene Ontology (GO) 'slim' terms [18], and the conservation score in mouse and K_A_/K_S _values between human and chimpanzee of dominant and recessive disease genes in each of the functional annotations were assessed (Table 4). Despite the variation in conservation values observed between the different functional classifications, we observe consistently higher conservation scores and lower K_A_/K_S _values (Table 4) in the set of dominant disease genes compared to the recessive disease genes. These results confirm that the evolutionary differences observed between dominant and recessive disease genes are due to different selective pressures acting on these two sets of genes during their history and not simply due to varying proportions of functionally dissimilar genes in each group.

In addition, when paralogues of dominant and recessive disease genes are similarly analysed, the higher conservation of dominant disease gene paralogues is evident in each functional group (Table 4).

### Evolutionary conservation rates by severity of disease

Analysis of the evolutionary constraints of disease genes, as indicated by human-chimpanzee K_A_/K_S _values, when categorised by the severity of disease displays a trend of reduction in selective pressure with decreasing severity of disease (Table 5). While this pattern is clearly evident in recessive disease genes, it appears to be more complicated in dominant disease genes. However, disease genes of both modes of inheritance that result in severe phenotypes (i.e. death before reproductive age) are more conserved than genes in which the consequent disease does not severely affect the reproductive fitness of an individual.

### Gene structure of autosomal dominant and recessive disease genes

Previously, it has been found that genes involved in hereditary diseases have different gene structure properties compared to the rest of genes in the human genome [2,19]. In particular, disease genes have longer coding sequences, more exons and more alternative splicing [19]. These specific sequence properties are thought to be due to the higher probability of suffering disease-causing mutations of genes that are longer and have more complex splicing patterns [19]. We analysed the structure of genes involved in autosomal dominant and recessive diseases to elucidate any differences between the two groups.

We have confirmed previous results, finding that the group of disease genes has, on average, a higher number of exons (D = 13.53, nD = 9.77), a higher number of alternative transcripts (D = 5.03, nD = 4.47), and a longer gene sequence (D = 62818 and nD = 52066) and coding sequence (D = 657.2 and nD = 509.6) compared to the complete set of human genes. All these differences have been tested using the Mann-Whitney and Kolmogorov-Smirnov tests and are significant. A similar analysis comparing the set of genes involved in autosomal recessive or dominant diseases shows no significant differences in the protein or gene length or in the number of alternative transcripts between these two sets of genes. However, there is a marginally statistically significant difference in the number of exons (DD = 13.65, DR = 14.63; p-value for M-W test = 8.5 × 10^-4^).

## Discussion

Our analysis reveals that the selective pressures on human autosomal disease genes involved in dominant and recessive disorders differ significantly. Dominantly-acting disease genes appear to be more conserved than recessively-acting genes. In addition, we have confirmed previous results showing that disease genes are generally more evolutionarily conserved than other human genes [2,5,6].

Previously it has been found that the proportions of different functional annotations in dominant and recessive disease genes differ substantially [1,14,15]. Therefore, it could be that the nature of the conservation pattern is determined solely by the function of the protein. However analysis of the conservation levels for the two groups of disease genes within each GO slim functional category (Table 4) would appear to refute this, as in all categories, dominant disease genes are consistently more conserved than recessive disease genes. Other factors have been shown to correlate with the protein evolutionary rate. In a recent report it has been shown that the age of a protein is inversely correlated with its evolutionary rate, that is, that older proteins evolve more slowly than proteins of more recent origin [20]. However, we have found that, in spite of the fact that recessive disease genes show higher evolutionary rates, they are better represented among old genes than dominant disease genes [see Additional file 3].

Overall, the results presented here show that the mode of inheritance of a gene is an important determinant of its rate of evolution. According to the nearly neutral theory of evolution slightly deleterious mutations may become fixed in populations [21]. Recessive genes should accumulate a larger number of such mutations than dominant genes, as in the former the mutant will be "hidden" from selection while heterozygous [22]. A higher fixation rate of slightly deleterious mutations in recessive genes would result in higher K_A_/K_S _ratios in this type of gene [4].

Analyses of the conservation levels of genes involved in recessive diseases have shown a significant decrease in frequency at high levels of conservation (high conservation score (Figure 1) or low K_A_/K_S _ratio (Figure 3C)). The most plausible explanation for this effect is that genes with high degree of conservation may be enriched for human essential genes, which cannot be involved in a recessive disease since a double mutation in them would be lethal. The analysis of RNAi phenotypes in *C. elegans *of human orthologues for the three groups of genes analysed supports this. We find that a slightly higher proportion of lethal genes in the set of dominant disease genes and a higher proportion of genes with no effect (WT) in the recessive disease set. However, we still find a considerable proportion of genes that are essential in *C. elegans *but classified as recessive disease genes in human. This may be due to the fact that, although the set of essential genes in *C. elegans *is likely to be enriched with human essential genes, there will also be cases in which the gene is essential in *C. elegans *but not in humans.

It has been suggested that essential genes should evolve at slower rates than non-essential genes [23]. Certainly, previous works have shown that essential genes in bacteria [24] and yeast [25] are highly conserved, although there have been some controversial results [26,27]. We have found that the level of conservation of human genes tends to be higher when the corresponding orthologue in *C. elegans *is essential than when it is not lethal. This supports the hypothesis that the sharp decrease of recessive disease genes at high conservation levels could be related to the paucity of human essential genes in the recessive disease set.

Therefore, it is possible that the greater degree of conservation exhibited by dominant disease genes vs. recessive disease genes is an effect of the higher likelihood of fixation of slightly deleterious non-synonymous mutations in the recessive disease genes coupled with an under-representation of highly conserved genes in this same dataset.

In addition, we analysed the conservation of disease genes according to the severity of the disease (Table 5). Our analysis reveals that the conservation of both dominant and recessive disease genes is highest in the category in which disease results in death before the onset of puberty, i.e. reproductive fitness equal to zero from a population genetics perspective. We acknowledge that the partition of disease genes into these rudimentary groups is a simplification, however, it serves to give insight into the relationship between disease severity and selective constraints.

We have also confirmed that disease genes show higher K_S _values, for both dominant and recessive genes, than the rest of human genes (Figure 3B, Table 3). Huang *et al*. reported similar results in an analysis between human and rat genes [3], and this phenomenon was also discovered in the analysis of the chimpanzee genome [6]. Furthermore, in this analysis the authors ascribe the higher K_S _level in disease genes to the more abundant presence of the mutation-susceptible CpG dinucleotide in disease genes. They report that when CpG dinucleotides are excluded from the analysis, the difference in K_S _rates between the two sets of genes becomes non-significant.

The tendency of non-disease genes to have closer paralogues than disease genes has previously been attributed to the fact that a close paralogue may be able to ameliorate the effect of a loss-of-function mutation in a gene by virtue of its similar functionality [16]. We find that recessive disease genes, which contain mainly loss-of-function mutations, have a deficit in close paralogues that could compensate for the loss of function. On the contrary, dominant disease genes, although possessing closer and hence more functionally similar paralogues, may not be reprieved from a disease state by the existence of close paralogues due to their dominant-negative effects. In addition, haploinsufficient genes (i.e. dominant disease) may be more dosage-dependent, in which case retention of paralogues may be advantageous [15]. However, the difference observed between the two groups of disease genes could also be due to less selective pressure on a recessive disease gene after gene duplication. Alternatively, gene duplications in dominant disease genes could be more recent than similar events in recessive disease genes. As can seen in Table 4, the difference in the conservation level of paralogues is not determined by protein function.

In this study we have focused on autosomal genes that are affected by recessively- and dominantly-acting mutations that lead to a disease phenotype. We readily acknowledge that we do not highlight other relevant groups of genes such as X-linked disease genes and imprinted genes [28,29]. However, cursory evidence of X-linked disease gene evolution is included [see Additional file 4].

## Conclusion

We highlight significant differences between disease genes, in terms of protein and DNA sequence conservation, paralogy and essentiality, when categorised by their mode of inheritance.

Our analysis reveals that genes affected by dominant disease mutations are more conserved than recessive disease genes. We attribute this to the fact that recessive mutations remain hidden from selection while heterozygous, which would allow recessive disease genes to accumulate a larger number of slightly deleterious mutations that eventually could become fixed in populations.

A number of studies have attempted to predict disease genes computationally using features such as sequence length, paralogy, sequence conservation, range of tissue expression, amino-acid composition and splicing signals among others [2,4,5,30]. These studies have treated disease genes as a homogeneous dataset. We suggest that the differences between dominant and recessive disease genes should be accounted for in future disease gene-prediction studies.

## Methods

### Data

Genes involved in hereditary disease are catalogued in The Online database of Mendelian Inheritance in Man (OMIM) [31]. We retrieved the list of genes from the 'morbid map' table in OMIM database. Using the NCBI LocusLink [32] database and the Ensembl database [33], we located the corresponding gene sequence records. The result is a list of 1647 genes associated with human disease. All other Ensembl protein-coding genes were classified as non-disease (nD). Disease genes were classified according to the mode of inheritance of the disease they cause using text mining automatic extraction from the clinical synopsis section in OMIM database and manual curation as reported in [14]. Genes were classified as autosomal Disease Dominant (DD; n = 498) if a mutation in a single allele has been reported as causative of a genetic disorder, autosomal Disease Recessive (DR; n = 662) if mutations in both alleles are required for the disease phenotype, and Others (e.g. X-linked diseases) as described previously. Genes in which both type of mutations (dominant and recessive) have been found were not used in the study.

### Calcultation of conservation score

Conservation score (cs) is an estimation of the divergence that has occurred between a pair of proteins during evolution, and is independent of the length of the proteins [2]. This score provides not only an estimation of the non-synonymous nucleotide substitution rate between a pair of proteins, but also takes into account the conservativeness of amino acid substitutions. Conservation scores were calculated using WUBLASTP (version 2.0) [34], which is based on the public domain NCBI BLAST version 1.4 [35]. Hits with E-values > 1 × 10^-10 ^were discarded. Smith-Waterman [36] alignment was performed on the pairs that gave a significant BLAST hit. The value of cs was calculated for each of the datasets DD, DR and nD using the relevant human protein-coding sequence in Ensembl version 34 as the WUBLASTP score of the closest homologue in the *Mus musculus *divided by the WUBLASTP score of the protein against itself, as reported elsewhere [37]. Similarly, the conservation score of paralogues was calculated as the WUBLASTP score of the closest paralogue divided by the WUBLASTP score of the protein against itself.

To assess the statistical significance of the results we computed two non-parametric statistical tests, the Kolmogorov-Smirnov (K-S) test and the Mann-Whitney (M-W) test, using the R statistics package [38]. To test whether there was a significant deviation from random expectation of the number of paralogues of dominant and recessive disease genes (X) at high levels of conservation (cs > 0.8) we randomly generated 10,000 datasets of proteins of identical sample size to the dominant and to the recessive disease gene datasets and counted in each set the number of genes with paralogues with cs higher than the threshold (0.8). Z-scores {Z_x _= (X - μ_x_)/σ_x_} and p-values {p_x _= Σ(n_x _≥ X)/N} were calculated (where mean = μ_x_, standard deviation = σ_x_, n_x _is the number of sets that score above the threshold X and N the total number of datasets)

### Calculation of nucleotide substitution rates at DNA level

The set of 13,454 human-chimpanzee orthologues used for gene evolution analysis by the Chimpanzee Sequencing and Analysis Consortium [6] was translated from Entrez gene entries to Ensembl gene IDs [33]. This dataset was merged with the set of dominant and recessive OMIM disease genes compiled earlier. This resulted in a non-redundant dataset of 886 disease genes, consisting of 367 dominant disease (DD) and 519 recessive disease (DR) genes, and 10,914 "non-disease" (nD) genes. Substitution rates K_A_, K_S _and K_I _data for the genes were taken from the Chimpanzee Sequencing and Analysis Consortium supplementary data [6]. K_A_/K_S _values for gene datasets were calculated by summing up over all non-synonymous and synonymous sites in each dataset as reported elsewhere [6]. Mann-Whitney and Kolmogorov-Smirnov tests were conducted using the K_A_, K_S _and K_I _data for each individual gene, and for K_A_/K_S _by excluding genes where K_S _= 0.

### Essentiality data in C. elegans

*C. elegans *genes were classified depending on their phenotype in RNAi experiments in three groups, Wild Type (WT), Lethal (L) or Disease (D). RNAi phenotypes were extracted from Wormbase Biomart [17]. We mapped the human orthologue of each *C. elegans *gene using orthologues pairwise data from Ensembl Compara [39]. The number of human genes corresponding to WT, L or D phenotypes in the RNAi experiments in *C. elegans *was counted for each of the groups analysed (DD, DR and nD).

### Evolutionary rates per functional classification

The dominant and recessive disease genes were classified according to the molecular function of each protein as determined by the Gene Ontology "slim" terms [18]. Protein conservation scores in mouse and paralogues were calculated as described previously. K_A_/K_S _values were calculated for each gene dataset by summing up over all non-synonymous and synonymous sites in each dataset.

### Evolutionary rates per severity of disease

Data on the severity of disease genes were obtained from Jimenez-Sanchez *et al*. [1]. In total 303 genes in our set were classified in one of the following groups according to disease severity: None, Mild (death >60 years), Moderate (death between puberty and 60), and Severe (death before puberty). K_A_/K_S _values for each group of genes were calculated as explained before.

### Gene structure analysis

Gene length, coding sequence length and number of introns were obtained from Ensembl database [33]. The number of alternative transcripts was obtained from Alternative Splicing Database [40]. Differences in these sequences properties between different groups (D, nD, DD and DR) were assessed using the Kolmogorov-Smirnov test.

## Authors' contributions

SJF carried out most of the analysis and statistical tests, participated in the design of the study and drafted the manuscript. MMA performed the gene age analysis and revised the manuscript critically. NLB conceived the study, participated in its design and coordination, and helped to draft the manuscript. All authors read and approved the final manuscript.

## Supplementary Material

Additional file 1 it contains supplementary figure 1. Click here for file

Additional file 2 it contains supplementary tables 1 & 2. Click here for file

Additional file 3 it contains supplementary figure 2. Click here for file

Additional file 4 it contains supplementary table 3. Click here for file

## Figures and Tables

**Table 1:** Molecular evolution and paralogy of disease genes involved in dominant and recessive diseases at protein level. Average values for mouse and paralogues conservation score for different sets of genes.

	**CS mouse**	**n**	**CS paralogues**	**n**
**Non disease**	0.730	16789	0.385	17962
**Disease**	0.795	1105	0.349	1108
**Disease Dominant**	0.810	474	0.420	478
**Disease Recessive**	0.783	631	0.295	630

**Table 2:** p-values for Mann-Whitney and Kolmogorov-Smirnoff test that asses the statistical significance of the differences between the averages and distributions of the groups.

**Mann-Whitney Test**					
	**CS mouse**	**CS paralogs**	**K_A_**	**K_S_**	**K_I_**

**nD vs Dis**	<2.2 e-16	3.38 e-04	0.170	1.36 e-10	0.260
**nD vs DD**	<2.2 e-16	5.24 e-04	6.37 e-04	8.34 e-07	0.567
**nD vs DR**	2.08 e-11	5.34 e-15	2.99 e-06	1.05 e-05	0.309
**DD vs DR**	7.59 e-06	4.44 e-16	7.17 e-11	0.212	0.798

**Kolmogorov-Smirnoff Test**					

	**CS mouse**	**CS paralogs**	**K_A_**	**K_S_**	**K_A_**/**K_S_**

**nD vs Dis**	<2.2 e-16	1.56 e-05	4.70 e-05	1.12 e-09	<2.2 e-16
**nD vs DD**	<2.2 e-16	8.70 e-08	7.98 e-05	3.06 e-04	<2.2 e-16
**nD vs DR**	<2.2 e-16	1.86 e-10	2.66 e-10	2.20 e-07	<2.2 e-16
**DD vs DR**	1.95 e-06	1.84 e-13	1.26 e-11	0.122	2.65 e-10

**Table 3:** Molecular evolution of disease and non-disease genes. Average values for K_A_, K_S_, K_I _and K_A_/K_S _for different sets of genes.

	**K_A_**	**K_S_**	**K_I_**	**K_A_**/**K_S_**	**n**
**Non disease**	0.00318	0.01400	0.01260	0.220	10914
**Disease**	0.00295	0.01644	0.01270	0.194	886
**Disease Dominant**	0.00260	0.01790	0.01274	0.157	367
**Disease Recessive**	0.00320	0.01540	0.01266	0.222	519

**Table 4:** Mean CS in mouse, in paralogues and K_A_/K_S _values for dominant and recessive disease genes by GO slim category

	**CS mouse**			
	**nD**	**Disease**	**Disease dominant**	**Disease recessive**

Enzyme regulation	0.778	0.790	0.818	0.755
Transcription regulation	0.800	0.860	0.871	0.824
Nucleic acid binding	0.795	0.835	0.851	0.803
Catalysis	0.809	0.819	0.832	0.815
Signal transduction	0.751	0.748	0.776	0.708
Structural molecule	0.828	0.831	0.838	0.815
Transporter	0.824	0.848	0.862	0.841
Carrier	0.838	0.881	0.884	0.880

	**CS paralogues**			

	**nD**	**Disease**	**Disease dominant**	**Disease recessive**

Enzyme regulation	0.374	0.272	0.300	0.236
Transcription regulation	0.353	0.422	0.440	0.359
Nucleic acid binding	0.358	0.370	0.431	0.250
Catalysis	0.381	0.355	0.430	0.331
Signal transduction	0.383	0.347	0.406	0.261
Structural molecule	0.449	0.482	0.595	0.207
Transporter	0.424	0.400	0.539	0.331
Carrier	0.425	0.432	0.571	0.402

	**K_A_**/**K_S _chimpazee**			

	**nD**	**Disease**	**Disease dominant**	**Disease recessive**

Enzyme regulation	0.163	0.233	0.223	0.251
Transcription regulation	0.170	0.144	0.109	0.253
Nucleic acid binding	0.188	0.164	0.149	0.190
Catalysis	0.185	0.179	0.150	0.189
Signal transduction	0.213	0.173	0.138	0.238
Structural molecule	0.201	0.183	0.155	0.250
Transporter	0.176	0.166	0.082	0.213
Carrier	0.170	0.197	0.123	0.213

**Table 5:** K_A_/K_S _mean values by mode of inheritance according to severity of disease: None, Mild (death >60 years), Moderate (death between puberty and 60), and Severe (death before puberty).

	*n*	*Disease*	*Disease dominant*	*Disease recessive*
None	99	0.186	0.133	0.224
Mild	36	0.167	0.114	0.215
Moderate	90	0.182	0.152	0.209
Severe	78	0.168	0.110	0.186
