# Supplementary material for: Differences in the evolutionary history of disease genes affected by dominant or recessive mutations
Source: BMC Genomics. 2006 Jul 3;7:165. doi: 10.1186/1471-2164-7-165 (PMC1534034; doi:10.1186/1471-2164-7-165)
Supplement: Additional file 1 — it contains supplementary figure 1. [file 1471-2164-7-165-S1.pdf]

Supplementary Figure 1

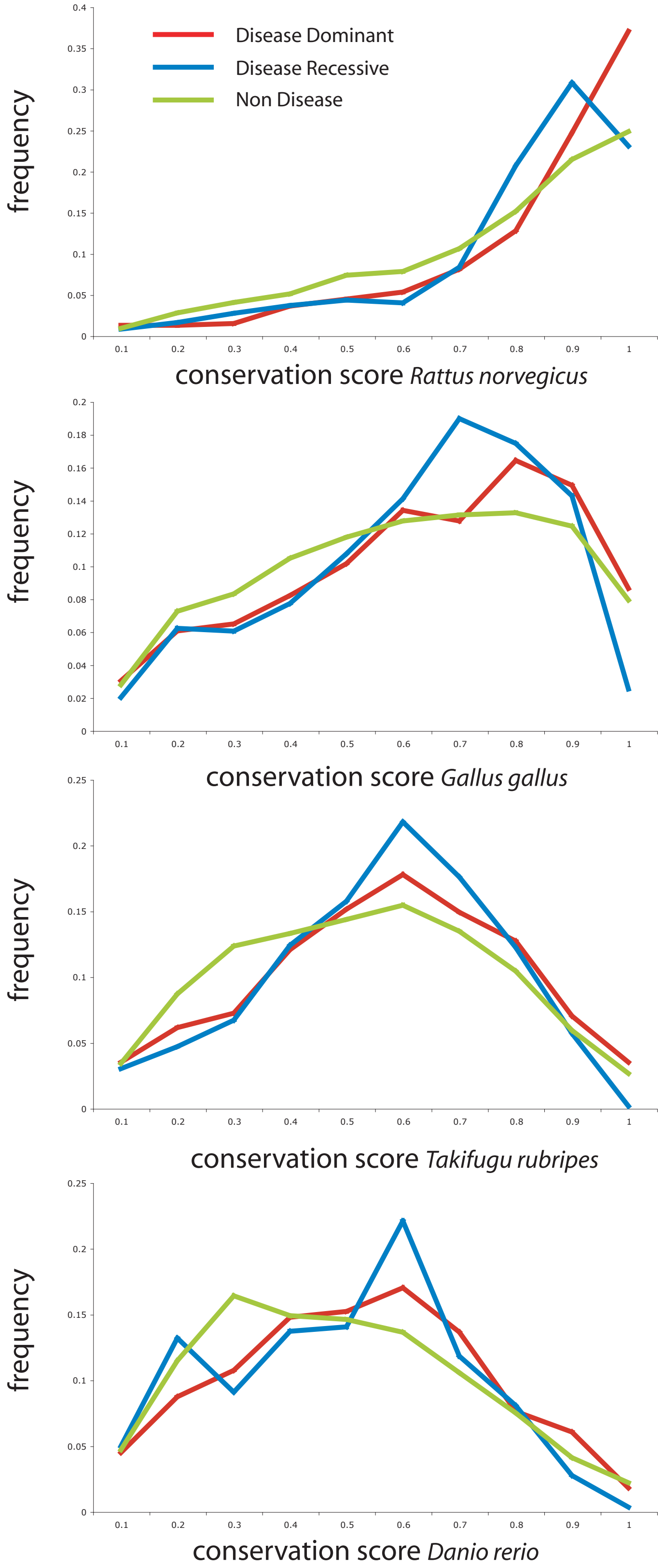

Supplementary Figure 1. Conservation of dominant and recessive disease genes at protein level. Distribution of conservation score in Rat, Chicken, Fugu and Zebrafish of dominant and recessive disease genes versus the rest of genes.
