# Supplementary material for: Differences in the evolutionary history of disease genes affected by dominant or recessive mutations
Source: BMC Genomics. 2006 Jul 3;7:165. doi: 10.1186/1471-2164-7-165 (PMC1534034; doi:10.1186/1471-2164-7-165)
Supplement: Additional file 2 — it contains supplementary tables 1 & 2. [file 1471-2164-7-165-S2.pdf]

Supplementary Table 1. Molecular evolution of disease and non-disease genes. Average values for K_A and K_S for different sets of genes. Nucleotide substitution rates for *Homo sapiens* (Hs), *Mus musculus* (Mm), and *Canis familiaris* (Cf) were downloaded from: www.broad.mit.edu/ftp/pub/papers/dog_genome/suppinfo/
Values are lineage-specific and were calculated from the dataset described in Lindblad-Toh *et al.,* 2005.

	Ka Hs	Ks Hs	Ka Mm	Ks Mm	Ka Cf	Ks Cf	n
Non disease	0.0239	0.158	0.0543	0.450	0.0319	0.241	10907
Disease	0.0237	0.170	0.0519	0.471	0.0305	0.254	791
Disease Dominant	0.0198	0.176	0.0441	0.469	0.0248	0.258	327
Disease Recessive	0.0264	0.166	0.0574	0.472	0.0345	0.251	464

Supplementary Table 2. Molecular evolution of disease and non-disease genes. Average values for K_A/K_S for different sets of genes. Nucleotide substitution rates for *Homo sapiens* (Hs), *Mus musculus* (Mm), and *Canis familiaris* (Cf) were downloaded from: www.broad.mit.edu/ftp/pub/papers/dog_genome/suppinfo/
Values are lineage-specific and were calculated from the dataset described in Lindblad-Toh *et al.,* 2005.

	Ka/Ks Hs	Ka/Ks Mm	Ka/Ks Cf	n
Non disease	0.171	0.126	0.140	10907
Disease	0.157	0.114	0.131	791
Disease Dominant	0.127	0.095	0.103	327
Disease Recessive	0.178	0.127	0.150	464

Lindblad-Toh K, Wade CM, Mikkelsen TS, Karlsson EK, Jaffe DB, Kamal M, Clamp M, Chang JL, Kulbokas EJ, 3rd, Zody MC *et al*: **Genome sequence, comparative analysis and haplotype structure of the domestic dog**. *Nature* 2005, **438**(7069):803-819.
