# Supplementary material for: Differences in the evolutionary history of disease genes affected by dominant or recessive mutations
Source: BMC Genomics. 2006 Jul 3;7:165. doi: 10.1186/1471-2164-7-165 (PMC1534034; doi:10.1186/1471-2164-7-165)
Supplement: Additional file 3 — it contains supplementary figure 2. [file 1471-2164-7-165-S3.pdf]

Supplementary Figure 2

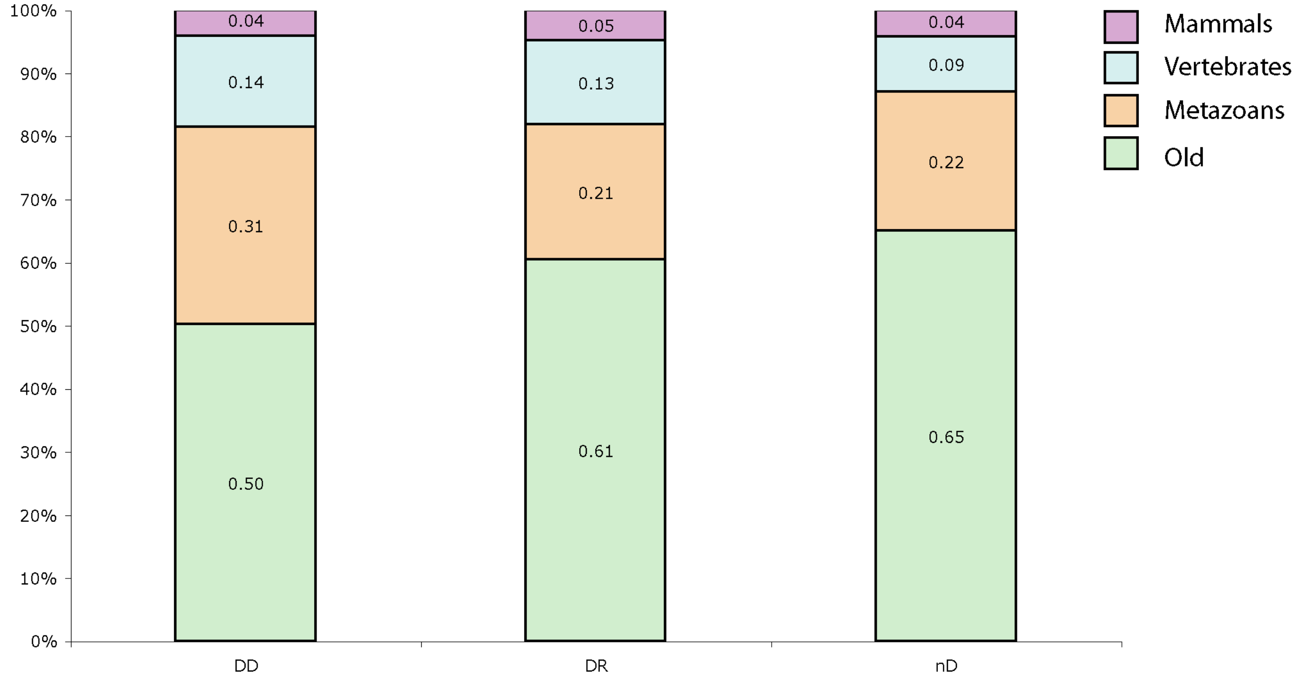

Supplementary Figure 2. Gene age analysis. Percentages of disease and nondisease genes in each of the gene age groups: Mammalian, Vertebrate, Metazoan and Old.

The genes used in this analysis were classified in different age groups - old, metazoans, vertebrates and mammals - by mapping through the Ensembl gene identifiers to the dataset in Albà and Castresana 2005. Briefly, in this dataset the age of human genes was estimated by performing BLASTP searches against the products of diverse eukaryotic complete genomes.

Alba MM, Castresana J: Inverse relationship between evolutionary rate and age of mammalian genes. Mol Biol Evol 2005, 22(3):598-606.
