# Supplementary material for: Differences in the evolutionary history of disease genes affected by dominant or recessive mutations
Source: BMC Genomics. 2006 Jul 3;7:165. doi: 10.1186/1471-2164-7-165 (PMC1534034; doi:10.1186/1471-2164-7-165)
Supplement: Additional file 4 — it contains supplementary table 3. [file 1471-2164-7-165-S4.pdf]

Supplementary Table 3. Conservation score of homologues in Ensembl proteomes.

	DH	DD	DR	DN	DX	nD
M.musculus	0.801418	0.82076	0.787852	0.775104	0.825667	0.766473
R.norvegicus	0.776439	0.789778	0.767027	0.750442	0.78285	0.743651
G.gallus	0.594214	0.601386	0.588961	0.579068	0.59085	0.582642
F.rubripes	0.52634	0.535832	0.519559	0.477308	0.53691	0.506372
D.rerio	0.46783	0.485242	0.45523	0.430523	0.461848	0.452645

DH: Autosomal hereditary disease genes.
DD: Dominantly-acting autosomal hereditary disease genes.
DR: Recessively-acting autosomal hereditary disease genes.
DN: Non-hereditary disease genes.
DX: X-linked disease genes.
nD: Non- disease genes.
